# Using the Power of Narratives in E-Learning for COVID-19 Vaccine Hesitancy Conversations: A Mixed Methods Study in Medical Education

**DOI:** 10.12688/mep.19767.2

**Published:** 2025-05-21

**Authors:** Aayushi Gupta, Anita Berlin, Graham Easton

**Affiliations:** 1 Institute of Health Sciences Education, Faculty of Medicine and Dentistry, Queen Mary University of London, London, England, UK

**Keywords:** narrative, e-learning, vaccine hesitancy, motivational interviewing, medical students

## Abstract

**Background:**

During the COVID-19 pandemic, we developed an e-learning resource to support medical students in having effective conversations with COVID vaccine hesitant patients (an urgent challenge). Development of information and communication skills elements was underpinned by narrative learning theory; learners interact with three fictional characters whose stories run through the resource in activities and role-plays. We co-developed the resource and characters with students, colleagues and local community.

**Methods:**

We used a mixed methods approach to evaluate the resource, including a survey of pre- and post- module self-confidence scores, and by thematic analysis of a focus group with seven final year medical students to explore their perceptions of how the story elements influenced their learning.

**Results:**

All students surveyed reported an improvement in their confidence in having effective conversations with vaccine-hesitant patients. The focus group analysis suggests that character-based narratives can promote learning online, particularly through engagement and accessibility, relatability of characters and their stories, improved memory, and emotional connection.

**Conclusions:**

This study suggests that character-driven stories have potential value in online learning about vaccine hesitancy conversations. Further research is needed to establish the nature of their impact on different aspects of learning including the duration of effect on students' communication skills and any patient-related outcomes.

## Introduction

**Figure 1.**
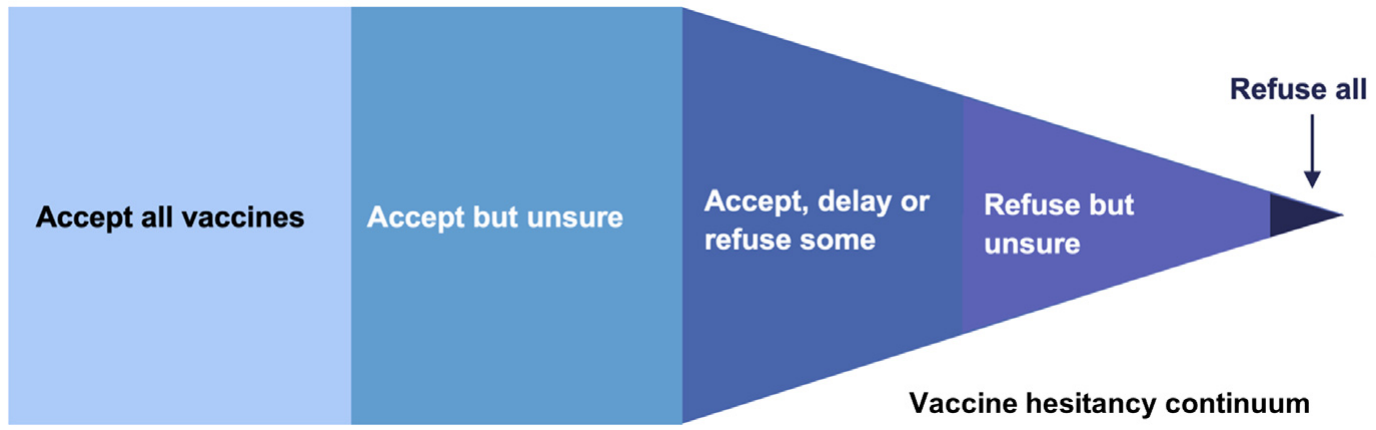
Continuum demonstrating attitudes towards vaccination. The central portion represents those who are deemed vaccine hesitant. Adapted from ( Butler, 2017).

Vaccine hesitancy is a well-established public health challenge ( MacDonald *et al.*, 2015; World Health Organisation, 2019), defined by the World Health Organisation as a “delay in acceptance, or refusal, of safe vaccines despite availability of vaccine services” ( MacDonald *et al.*, 2015). Attitudes to vaccination can be viewed as a continuum, from those who accept all vaccines to those who actively oppose all vaccines (see Figure 1).

Causes of vaccine hesitancy are complex and context-specific, and it is thought to be influenced by issues such as perception of risk, low confidence in the vaccine, access barriers, inconvenience, and lack of endorsement from trusted providers and community leaders ( Scientific Advisory Group for Emergencies, 2021). COVID-19 vaccine hesitancy remains prevalent in our local East London community and is disproportionately high in ethnic minorities and religious groups living in East London ( UK Health Security Agency, 2022).

### Development of an e-learning module

To address this problem, we designed an e-learning module to support healthcare professionals and medical students in having effective conversations about vaccine hesitancy – an urgent and ongoing challenge during the pandemic ( Razai *et al.*, 2021b). Many of our medical students were having these conversations with patients on their clinical placements yet had had no specific training. We designed the module with three main principles in mind:

(1) **Online** delivery, allowing students to complete it asynchronously from anywhere in the world, in a self-directed fashion, providing flexibility, accessibility and comfort ( Mukhtar *et al.*, 2020). With the shift to online teaching through the pandemic, it was particularly important for this to be an online resource.
(2) **Motivational interviewing (MI)** as the underlying counselling approach. MI is a recognized technique used in behavior change, and has a strong evidence base ( Gagneur, 2020; Lundahl *et al.*, 2013; Rollnick *et al.*, 2005; Rollnick *et al.*, 2010). MI promotes a *“guiding”* approach in vaccine hesitancy consultations, which is likely to have more meaningful outcomes than a didactic, *“telling”* approach ( Easton, 2021; Lewandowsky *et al.*, 2021; Razai *et al.*, 2021a). In MI, the goal of the healthcare professional is not to persuade or coerce patients, but to ensure they have reliable, relevant information and to support them to make their own decision ( Lehner *et al.*, 2021).
(3) Development of learning materials was underpinned by **narrative learning theory**: *“Fostering learning through stories”* ( Clark & Rossiter, 2008). Three characters’ stories would run through the resource in activities and role-plays. The literature suggests that narratives are a potentially powerful education tool that can be useful in promoting memory, engaging learners through emotions, and providing relevant context to learning ( Easton, 2016). Despite the theoretical promise, there is limited evidence for the benefits of narrative learning approaches on long-term learning outcomes, clinical behaviour or patient outcomes. For example, a systematic review of digital storytelling (which combines traditional storytelling with digital tools) showed minimal impact of patient stories alone on health professionals learning ( Moreau *et al*., 2018). Developing models for narrative medicine education and integrating and evaluating diverse storytelling formats, such as the digital storytelling approach we developed in this project, could further enhance student learning experiences

Previous work has explored reasons behind vaccine hesitancy and identified groups within society with higher rates ( Aw *et al.*, 2021; Osama & Majeed, 2021; Robertson *et al.*, 2021; Sallam, 2021; Troiano & Nardi, 2021). Health communication around vaccines is often more accessible if it is tailored to these different population groups ( British Islamic Medical Association, 2021). We wanted to reach groups within our community who have higher rates of vaccine hesitancy, including minoritised ethnic groups. We collated a range of resources for the e-learning module, as a collaboration with library information specialists, clinical academics, and the local community and council, in order to prepare current and future healthcare professionals to have productive conversations about vaccine hesitancy and explore common patient concerns.

To portray our characters in the e-learning module, we used a “thick narrative approach” (thorough case description and media-rich resources) that has been shown to increase teaching efficiency within online medical education ( Bizzocchi & Schell, 2009). Genuine stories tend to be more influential, so we wanted to provide this by giving each character an emotional dimension and a story ( Bowmaker, 2022). Narratives provide students with a holistic view of their patients, especially with the use of first person. It has also been shown that narratives can increase student enthusiasm and, in doing so, learning and engagement ( Lindgren & Mcdaniel, 2012).

### Evaluation aims and research questions

For this evaluation, we were particularly interested in understanding more about the novel narrative elements of our e-learning module. Our aim was to find out how students experienced the narrative elements of the resource and how it influenced their learning and confidence in having vaccine hesitancy conversations with patients. Our research questions were:

- To what extent and in what ways did the character-driven narratives influence learning of medical students who used the resource?
- What effect, if any, did the resource have on medical students’ self-confidence rating in relation to having conversations with COVID-19 vaccine hesitant patients?

## Methods

### The e-learning module design

**Figure 2.**
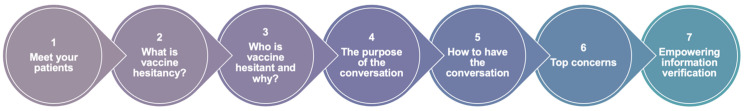
The seven sections of the online resource on vaccine hesitancy.

The e-learning module was designed to take 60–90 minutes to complete. The material was hosted on the university’s online virtual learning platform, allowing asynchronous completion of the module. For this study, our main learner group was medical students (although other health professionals and community workers later made use of the module). The resource was developed by GE and AG and received input from public health staff at one of our local borough councils, Tower Hamlets. The overview of the resource is outlined in Figure 2. Each section had learning objectives and ended with a summary. Interactive quizzes featured throughout the resource.

**Figure 3.**
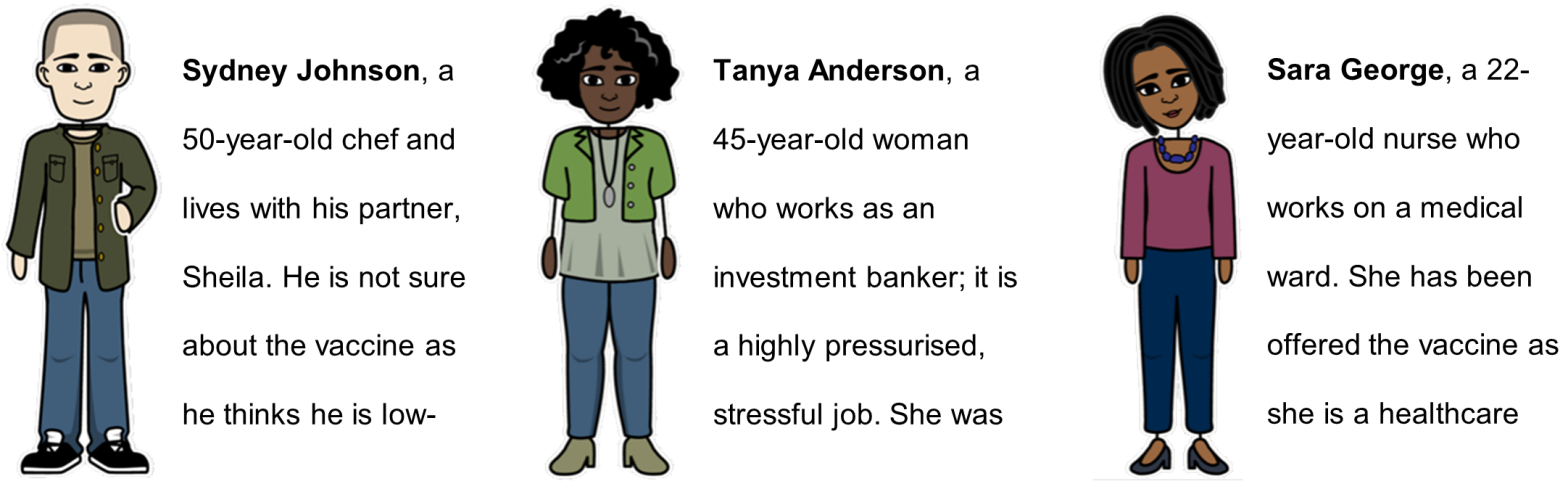
The three characters that students followed throughout this resource, with their introductory statements. Created with Storyboard That.

We developed the module using several different types of media including Storyboard That, H5P, Microsoft PowerPoint (Version 16.46), and video. The module introduced users to the stories of three characters early on (see Figure 3). These characters were revisited throughout the materials allowing students to explore each character’s story arc via interactive storyboards and through roleplay. Characters were portrayed as cartoons to make the resource media-rich, aiming to increase engagement. Each character’s scenario was chosen to reflect the concerns and demographics of the local community. Alongside our own clinical experiences, the characters were co-developed with a local primary care doctor to make them as realistic as possible.

Additionally, one of three characters, Tanya, was portrayed by an actor in two video roleplays included within the resource. These portrayed an “effective” and “not-so-effective” patient consultation discussing vaccine hesitancy to demonstrate the impact of motivational interviewing. This video was interactive, pausing and asking students to answer questions on the roleplay as the consultation progressed. At the end of the video, the actor gave feedback on each consultation; what worked well and what the doctor could do to make the conversation more effective.

### Evaluation methods

We chose a mixed qualitative and quantitative approach to answer the research questions as we wanted to explore: (a) student perceptions and engagement with the characters’ stories, and (b) the impact of the module on self-reported confidence in conversations with vaccine hesitant patients. The qualitative approach used focus group interviews. The guiding theory for our evaluation was the same as for the resource design – narrative learning theory. Narrative learning theory is an attempt to explain how narratives might promote learning. The central proposal for narrative learning theory rests on our strong impulse to make meaning from our experience by creating stories or narratives ( Hopkins, 1994). Learners connect new knowledge with lived experience and weave it into existing narratives of meaning ( Rossiter, 2002). Narrative learning theories therefore fall under the broader umbrella of constructivist learning theory, in which learning is seen as construction of meaning from experience ( Dewey, 1926).

We adopted three methods of evaluation, with the same group of seven students who had completed the pilot module:

(1) Focus group of seven students
(2) Pre- and post-module self-confidence scoring
(3) General feedback (optional) as part of the module

### Sampling

Purposive sampling was used to recruit final year medical students before making the module available to all students. This cohort had completed their final examinations so had time for the study, and their involvement in the module would not give them any advantage over fellow students. They were also more likely to be in a position where they would be having vaccine hesitancy conversations. We aimed to recruit between seven and 10 students as recommended for an effective focus group discussion ( Kuper *et al.*, 2008).

### Recruitment

To recruit students, an email was sent to all MBBS final year students at Queen Mary University of London via the President of the Students’ Association. Students replied to express interest on a first come first served basis. Inclusion criteria were being a final year medical student; completion of the consent form; completion of the module; ability to attend the focus group. To incentivize participation, completion of the module, confidence assessment and focus group allowed students to apply for an award which formally recognizes student participation in co-development of educational resources. Seven final year students were recruited from the medical school.

### Data collection

The data collection period was from 14/6/21 – 28/6/21.

***Virtual focus group.*** We gathered data through a virtual focus group after the medical students had completed the online module independently. The focus group was held online via Microsoft Teams to maintain social distancing. AG facilitated the focus group. The group ran following standard focus group methodology ( Kuper *et al.*, 2008), establishing an informal and conversational environment and encouraging participation from quieter members of the group. It was recorded and audio was later transcribed by AG. AG took notes during the discussions of recurring ideas and concepts. The focus group ran for one hour. AG asked open questions based on an interview topic guide ( Gupta *et al.*, 2023e), derived from the theory underpinning the work, specifically regarding the use of stories and the impact of the characters. A transcript ( Gupta *et al.*, 2023b) was created from this audio recording to be analyzed.

***Confidence rating scores.*** Students were also asked to submit self-confidence scores (Likert scales out of 10) ( Gupta *et al.*, 2023a; Gupta *et al.*, 2023c; Gupta *et al.*, 2023d), pre- and post-module completion via an electronic survey. Students were asked “How confident do you feel about having effective conversations with vaccine hesitant patients, on a scale of 0-10 where 0 is not at all confident, and 10 is totally confident?”.

***Free-text feedback.*** Additionally, an optional free-text feedback form featured at the end of the module. This consisted of a single rating question using a Likert scale (“ *Overall module rating, out of 10*”) then two free-text questions: (1) *“What did you really like about the module? Any specific sections or approaches?”*, (2) *“What could we do differently to make it better?”*.

### Analysis

The data analysis period was from 1/8/21 – 31/8/21.

Thematic analysis of the transcript was undertaken alongside review of the notes made during the focus group following steps outlined in previous work ( Braun & Clarke, 2012). AG read the data line-by-line to manually code topics throughout the text and identify patterns of meaning. AG and GE independently reviewed and reflected on all the initial codes and quotes to group these into emerging themes. After further discussion, merging and rearrangement AG and GE identified six coherent themes relating to the module’s accessibility, the use of characters/stories, and their pedagogic value that we describe in the results below. Both authors agreed on the final themes that emerged from analysis.

## Ethics

Ethical approval was granted by Queen Mary Ethics of Research Committee (reference number QMERC20.400, approval date: 04.06.2021). Students were given a Participant Information Sheet beforehand and signed a written consent form if agreeable. Students were given the opportunity to re-review the details of the consent form and ask any outstanding questions prior to the focus group discussion starting. Ground rules were established: reassurance was given that AG played no direct role in student assessment; students were requested to keep the details of the focus group discussion confidential; cameras were encouraged to be turned on to facilitate more authentic discussions; any individual affected by triggering topics would be directed to student support services. Students were allocated a participant number beforehand, which AG and students used to refer to one another rather than student names. This enabled the audio to be recorded with consent and suitably pseudo-anonymized for storage, with a view to destroy data once this project is complete. To ensure participation in this study did not compete with students’ personal study time or scheduled teaching, the focus group did not run during formal teaching time and was only offered to those students who had completed examinations.

## Results

Results were collected in the form of: (1) analysis of focus group transcript for key themes; (2) pre- and post-module self-confidence scores; (3) optional anonymized feedback from students. Of the final year students who received the study recruitment email, around 2% replied to express interest. All students were eligible and participated in the study. Of the seven students, two identified as male (29%) and five as female (71%). Whilst we did not formally collect demographic data on ethnicity, overall we felt the make-up of the focus group broadly reflected the diversity of the medical student body.

In the pre-course survey about students’ confidence and experience of having vaccine hesitancy conversations, three reported no experience at all, two reported very little experience, two reported some experience, and one reported quite a lot of experience. Some students in the focus group said they had vaccinated more than 100 patients. In terms of self-reported confidence having these conversations, on a scale of 0–10 where 0 is not at all confident, and 10 is totally confident, two students scored 2, three scored 3, one scored 4, one scored 5, one scored 6 and one scored 8.

### Key themes from focus group analysis

***Engagement and accessibility.*** The characters in the module engaged learners, made learning accessible as chunks of text felt less intimidating, and held students’ attention. Re-visiting the characters throughout the resource grounded the module. Students appreciated that the characters were a core stem that information kept referring to, with reports that learning is much easier with real patients to contextualize the information, which echoes the literature ( Perper, 2019). The stories gave context and invited the audience to engage and co-create through their imaginations.

Student 1: *Learning is much easier with real patients to hang knowledge off... [rather than being told] people generally may not want the vaccine.*

***Relatability of characters and their stories helped learning.*** Evaluation shows students drew parallels between real-life conversations and the characters they met in this resource. Most junior doctors and medical students will be having these conversations and this resource and the characters’ stories helped to frame these conversations. In fact, some students in the focus group had vaccinated over 100 patients at the time therefore recognized the vaccine hesitancy concerns in this module. Others simply sympathized with the concerns because of conversations with family and friends. The characters were familiar, relatable, and authentic, particularly the hesitant healthcare worker.

Student 5: *I liked having the characters. If you meet a patient with a condition, it really sticks in your head a lot more than just book learning about the condition. It kind of brings it to life.*
Student 4: *You can definitely draw parallels from people I have met in real life.*

One student highlighted the value of the video consultation, where one of the cartoon characters was brought to life by a “real” actor:

Student 6: *Cartoons didn’t really bother me until we got to the videos with the actor playing Tanya, I think that would be hard with cartoons alone. I don’t think the cartoons were a drawback, the videos and cartoons added to each other, but I related a lot more when I saw the interaction between real people play out.*

***Emotional connections.*** By understanding each character’s background, students were able to develop empathy for these characters and the challenges facing them. Interestingly, students reported Tanya generated the strongest emotional response, perhaps as she was portrayed “in real life conversation” by an actor in the video consultations. Students suggested that the video brought this character to life: hearing the emotions in her voice, and non-verbal factors such as her facial expressions, made Tanya more relatable than the characters purely portrayed via text and cartoons. Students did report that the cartoon depiction increased relatability versus having no visual representation. We discussed the possibility of using photographs of real people instead of cartoons; even if not all characters had a video, perhaps even a photograph may aid learning.

Student 3: *I really liked the videos because when you hear Tanya speaking, you can hear all the emotion in her voice, and you can hear the concern. You can see her facial expressions. You can see there is a lot of worry and anxiety in what she is saying. It’s the sort of thing you can’t really get from reading text, because you’d read it in your own voice and in your own way, but when it’s happening like this then it’s more unique and distinct which helps you remember it a lot more.*
Student 2: *Seeing the non-verbal and observing the interaction between two individuals, and placing yourself in that position, is what makes the video so much more relatable. If you were to make a cartoon video, unless it was high quality, you wouldn’t get the emotion and non-verbal in the same way that you do when it’s an actual person who is an actress, and a really good actress at that.*

**Figure 4.**
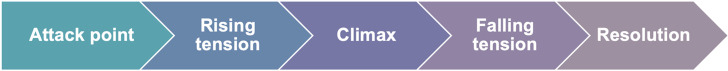
A story arc can facilitate scientific flow, starting with information to pique and maintain interest, then a climax, followed by a satisfying ending. Adapted ( Perper, 2019).

***Memory boost.*** Students felt stories helped them remember each character, activating memories of previous consultations and providing a framework to build knowledge on. The degree of this memorability was variable depending on the media used: videos seemed to be more effective than pictures, which were more memorable than no pictorial depiction. The power of the story arc also increased memory through enhanced engagement (see Figure 4) ( Perper, 2019). Characters featured throughout: in the introduction (where students rate each character on a vaccine hesitancy scale) and later in roleplays and the e-learning conclusion. Revisiting each story line in a temporally spaced manner allowed consolidation of learning. Finally, providing an outcome for each character was important because it gave closure to the students who had invested time and emotion into each narrative, allowing them to appreciate the outcome of an effective MI consultation.

Student 2: *Definitely once I saw Tanya in the video, I remembered her case a lot more clearly and I felt like I could relate a lot more to what she was saying. and even now, thinking back to the other two cases, I can’t remember as much their reasons as I do Tanya’s.*

***More stories, more learning?*** Another consideration was the number of characters included within the resource. When designing the resource, we wanted to demonstrate the diversity within vaccine hesitant groups without producing too many characters, which risked overwhelming students who might lose any empathic connections. During the focus group, there was mixed feedback. Generally, students felt that for the length of the resource, there was an appropriate number of characters but perhaps there could be a few extra optional case studies after completion of the module.

Student 2: *Obviously it’s a balance between a module that takes too long to complete where people start just clicking through because they’re frustrated that it’s taking so long but we probably could have benefitted from 1–2 more characters with the same level of detail and perhaps some additional optional characters at the end with a different view or perhaps a different outcome and how you could approach these.*
Student 6: *I thought 3 was a good number to avoid information overload but I like the suggestion of optional extra characters.*

***What if no story…*** Students reported that without the stories utilized in this resource, they would have struggled to maintain focus whilst completing the module. Moreover, an interest in the ending of each story motivated students to want to complete the resource, giving a sense of resolution. Finally, it would have been more difficult to recall learned information without the characters to contextualize the information.

Student 7: *I was definitely invested in the stories, from the introduction all the way to the conclusion. It’s good to have an ending because it gave closure.”*
Student 6: *“I did find them [the characters] really helpful.*

### Pre- and post-module self-confidence scores

**Figure 5.**
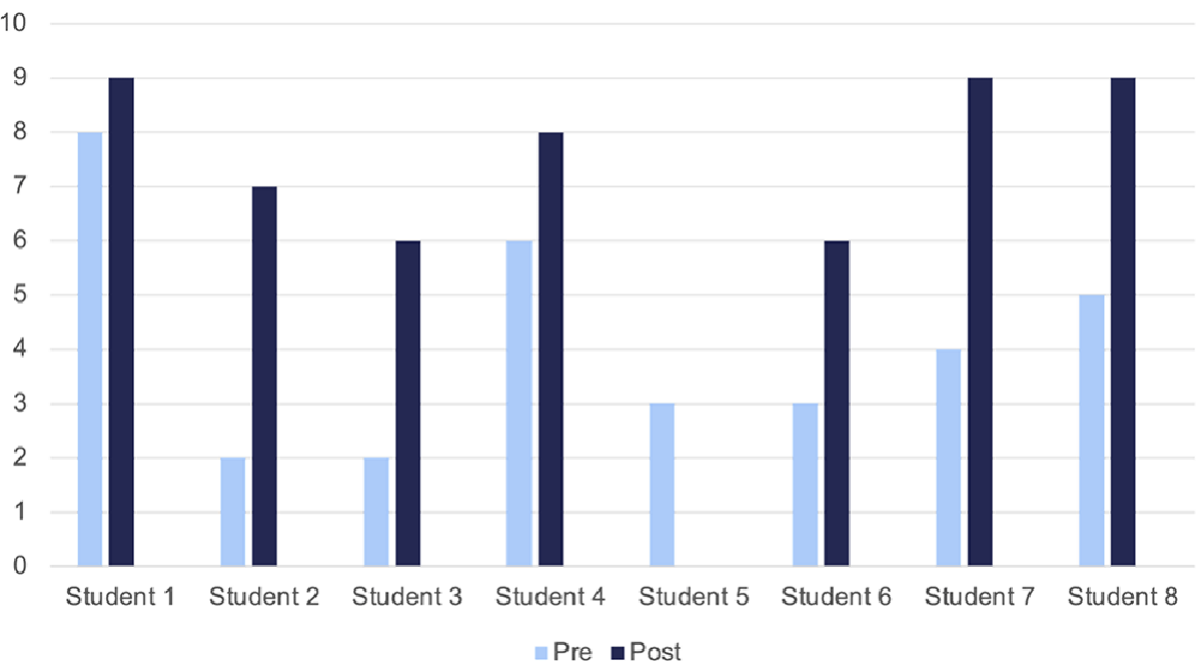
Pre- and post-module self-confidence scores (out of 10). These scores were submitted online as part of the module.

Students were asked “How confident do you feel about having effective conversations with vaccine hesitant patients? On a scale of 0–10 where 0 is not at all confident, and 10 is totally confident?” (see Figure 5). Student 5 did not submit a post-module score. Given that seven students participated, possibly a student accidentally submitted a pre-module score twice, most likely students 5 and 6 represent the same student.

These results demonstrate a mean improvement of +3 points on the scale. All students reported an improvement in their confidence, ranging from +1 point (n=1) to +5 (n=2). Table 1 demonstrates how previous experience in speaking to vaccine hesitant patients affects improvement in confidence. Students were asked “How much experience would you say you have in talking with vaccine hesitant patients?”. This table demonstrates there was greater self-perceived improvement in those with no or very little experience.

**Table 1.** Average change in confidence pre- and post-module, out of 10, stratified by self-reported experience in having conversations about vaccine hesitancy.

Experience	Average improvement in self-confidence
No experience at all	+4
Very little experience	+5
Some experience	+3
Quite a lot of experience	+1

### Optional anonymized feedback

**Figure 6.**
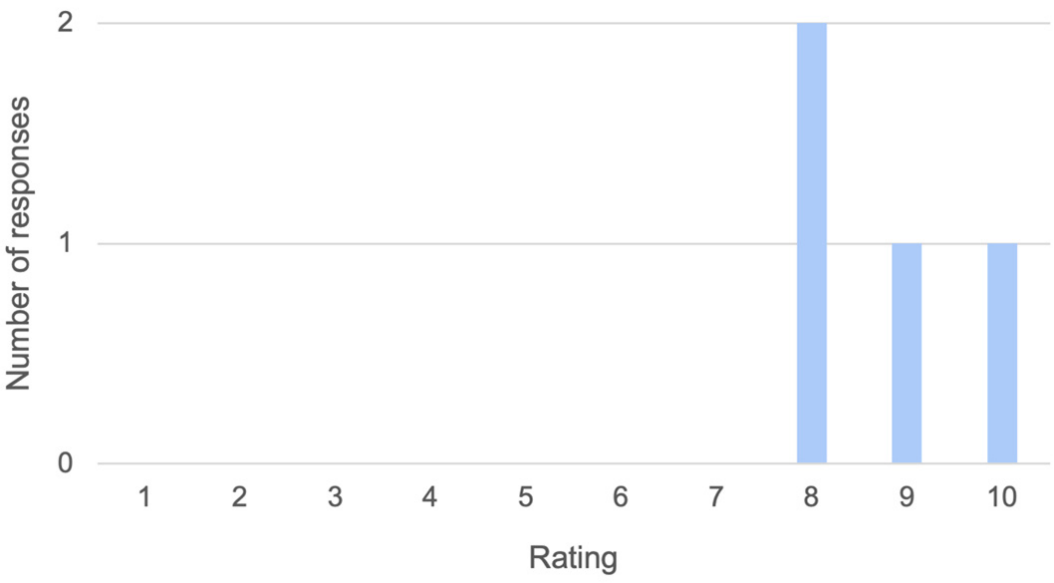
Student feedback on overall module rating (out of 10). These scores were submitted anonymously through an online survey.

Of the seven students who finished the module, four of them (57%) completed the optional online feedback, comprising of three questions. Firstly, “How would you rate the module overall, out of 10 [where 10 is outstanding and 1 is very poor]?” (see Figure 6). Responses to questions two and three are tabulated below (see Table 2). These comments show that Tanya was particularly memorable, as she is the only named character in the feedback. Feedback on potential improvement in module interface and navigation was also collected, but further discussion is beyond the scope of this paper.

**Table 2.** Student responses to optional questions, submitted anonymously online.

What did you really like about the module? Any specific sections or approaches?	What could we do differently to make it better?
*Good variety of resources, helpful ways to address vaccination myths,* *interesting to read about vaccine hesitancy in certain community* *groups and why that might be.*	*The interface could be more user friendly, maybe more interactive* *with quizzes etc*
*I liked that the module gives you very practical advice for* *approaching these conversations and also highlights specific* *knowledge that can be used in conversation with patients. The* *opportunity for roleplay is very useful*	*Overall, I feel it was a great module. A huge variety of content. Very* *engaging, lots of think about.*
*I liked the use of external articles and resources. I liked the examples* *of good/bad techniques. I appreciate the option to complete a* *number of sections and come back to the course.*	*Make sure the roleplay section can be utilized fully – I could not do it* *as I had no one to practice with and thinking about responses is not* *as helpful*
*Really comprehensive, really enjoyed the two videos with Tanya – the* *first video is shockingly what I see on placement and can agree is not* *a very effective method at all for discussing concerns with patients!* *These videos were helpful for me.*	*I would always advocate for in person teaching over online. I realized* *part way through that I was clicking through the tasks but missing a* *lot of the written information available on the first page, so I had to* *go back through*

### Limitations

There are some limitations of this study. Whilst qualitative studies do not necessarily need large sample sizes to allow collection of in-depth insights, perhaps having two focus groups would have demonstrated a broader range of views. As we used a convenience sample (the challenges of conducting the study in lockdown during the Covid pandemic and limited availability of medical students, meant we only used a single focus group), we cannot be sure how closely our focus group students represent the wider student population, and we should consider volunteer bias ( Heckman, 1990). Data shows volunteers in medical education tend to be higher achievers within their cohort, with women and ethnic minority groups being less likely to participate ( Callahan *et al.*, 2007). This affects how translatable the results from this study might be to the entire student cohort. Low levels of interest in study participation are likely multi-factorial, including email and survey fatigue, and reluctance to engage once finals examinations were complete.

Focus groups have well established limitations, whether that is the dominating individual or the potential for conflict between group members ( Smithson, 2000). Participants may not feel free to express their views, depending on their personality type, and certain sensitive topics are automatically harder to discuss in a group setting, particularly virtually.

 It could be argued that the facilitator for the focus group should have been an impartial individual, as students were aware that AG helped to develop the resource. Guidance recommends that the facilitator should maintain neutrality to avoid bias in participants responses ( ACET Inc, 2011). Although we took care to encourage negative feedback, and students did not seem hesitant to offer these, it is possible students did not contribute all their negative opinions so as not to offend AG. Similarly, there is a risk of moderator bias from accidental leading. However, in mitigation, AG was only minimally involved in developing the actual resource [GE oversaw most of this], we set clear ground rules about encouraging honesty, clarity that AG would not be involved in any of their formal course assessments, and we ensured another author [GE] also independently analysed the data.

## Discussion

This work highlights the potential power of narratives in medical education, specifically when used in online asynchronous modules. The results suggest that narratives can promote learning online, particularly through improved memory, relatability, and emotional connection. Our themes echo existing literature on the use of narratives in medical education ( Charon, 2006; Greenhalgh, 2001; Hunter, 1993).

Stories are used in various ways in medical education; for example, during ward rounds and clinical case discussions to illustrate diagnostic reasoning and decision-making ( Hunter, 1991), or in narrative medicine courses and medical humanities programmes which often use patient stories to foster empathy and reflection ( Milota *et al*., 2019). Digital storytelling (which combines traditional storytelling with digital tools) is increasingly used to promote patient-centred care with patient narratives offering insights into lived experiences ( Moreau *et al*., 2018).

There is some limited evidence demonstrating the impact of narrative-based approaches on learning in health professions education . For example, narrative medicine, as explored in a systematic review, has shown positive changes in students’ attitudes, perceptions, attainment of new knowledge and skills, behaviour, and improved awareness of patients’ perspectives ( Milota *et al*., 2019). Storytelling has been shown to prepare students for uncertainty in clinical practice ( Papanagnou *et al.*, 2021), and can be an effective way for students to learn about medical ethics ( Paton & Kotzee, 2021). In classroom lectures, narratives can provide context, engage learners, and aid memory retention by connecting new knowledge to lived experiences ( Easton, 2016). Digital storytelling has been shown to improve medical students’ critical thinking ( Zarei *et al.*, 2021), although a systematic review of digital storytelling showed minimal impact of patient stories alone on health professionals learning ( Moreau *et al.*, 2018).

The use of stories in this e-learning resource seemed to help medical students by achieving its aim to help frame vaccine hesitancy conversations through motivational interviewing. By creating relatable and recognizable characters, students appreciated following a story arc throughout with a sense of satisfaction that came from reaching a resolution for each character. It was important to us to assess the impact of applied narrative learning theory in e-learning, given the shift towards virtual learning in undergraduate medical education during the pandemic ( Gill *et al.*, 2020). Previous work has established a role for narratives in in-person synchronous teaching, aiding medical students by providing context to learning, increasing engagement, and improving memory ( Easton, 2016). Stories can easily be shared in the form of case studies or shared experiences via patient or clinician narratives. This current work suggests that stories, in the form of character-based cartoons and videos, can also be effective tools to support online learning.

The students in our study felt that the characters’ stories made them easier to relate to and engage with. Narratives and story structure may help to engage learners by offering a connection with existing knowledge and experience, and by making the unfamiliar familiar. For example, Holt and others have shown that story structure can help learners call up existing banks of knowledge and can make new information seem relevant ( Holt, 1995; Pinker, 1995).

Students also highlighted that the stories were easier to remember, a key element of the learning process. Fernald’s study of undergraduates suggested that they found stories more memorable than formal book or lecture descriptions ( Fernald, 1987). Classical stories are defined by Haven as:

A detailed, character-based narration of a character’s struggles to overcome obstacles and reach an important goal *-* ( Haven, 2007)

These classical stories might be particularly memorable because they involve the listener in the actions and intentions of believable characters. Our study seems to support this idea with students all agreeing that the characters helped them to remember previous experiences, scaffolding learning and building new learning from those prior experiences, all key facets of constructivist learning ( Dewey, 1926).

Stories engage learners by involving them and encouraging an empathic response – the details of characters and their motivations draw us into a story and urge us to take the character’s perspective ( Rossiter, 2002). Students in this study felt that the characters’ stories allowed them to engage empathically, which in turn made the stories more relatable, engaging, and memorable. It would be interesting to explore whether this promoted any greater sense of empathy for patients the students subsequently spoke with. It also raises the question that if we want to use authentic and relatable stories which promote an emotional response in learners, could we not employ real people and patients instead of cartoons or actors? Would the emotional connection be even greater in that case?

Taking this resource forwards, to increase accessibility, non-students will be able to access the page with a password, allowing wider dissemination of the materials to healthcare professionals. Similarly, providing this resource in other languages would be the next consideration. There is also a lot of scope to further develop the resource itself *e.g.,* addressing the technical issues highlighted by students. Perhaps, further empathy and memory could be promoted by adding voice recordings of the other two characters: Sydney and Sara, who do not presently have a “real-life” depiction by an actor. It would be interesting to assess whether giving these cartoons a voice would increase emotional connection. Evidence shows students appreciate information in audio form, but when delivered exclusively this way, it is associated with poorer exam performance ( Daniel & Woody, 2010; Furnham, 2001). A compromise would be to provide both audio and written information alongside the existing visual media. The longer-term implications of using videos as a resource must also be considered. To generate and maintain empathy and relatability, the characters and their stories must be believable ( Baron *et al.*, 2019). This requires money to pay for the production costs, including actors. Without this, the believability may be challenged. The resource is interactive, but students are presently unable to alter the path their character takes. By considering branching storylines, students may take greater ownership over their decisions. This would have to be balanced against not overcomplicating the storylines to maintain accessibility ( Pulse Learning, 2015).

The evaluation of this resource primarily took place via subjective measurements, both qualitative (focus group interviews) and quantitative (surveys) ( Goldie, 2006). In the future, more objective styles could also be considered ( Morrison, 2003). However, to answer this research question, we believe that qualitative data was most valuable. We also think it would be important in future studies to include stronger outcome measures, particularly those relating to the patient perspective and the enduring effect on medical students’ communication skills or empathy, guided by outcome measures proposed by Kirkpatrick ( Kirkpatrick *et al.*, 1967) and others. Another important measurement would be patient-reported outcome measures (PROMs): interviewing real-life vaccine hesitant patients who have had consultations ( Kirkpatrick *et al.*, 1967). These could be split into two groups, with clinicians split between either having completed the vaccine hesitancy resource or not. Confounding factors would have to be accounted for such as clinician experience, specialty, demographic. This would allow for fair comparison.

When developing the resource, we were aware of the risk of stereotyping. We tried to mitigate this risk by gathering feedback from local community groups and students. The students felt the characters did not stereotype groups of patients and echoed authentic patterns in the local population. The first iteration of this module received input from the Public Health Program Manager of a local health authority in Tower Hamlets. The feedback advised adding more case studies, reconsidering the language used and potentially reordering content. To avoid the risk of inadvertent racial bias and profiling by students, adding more Caucasian characters could help. A suggestion was made to use “rumors” instead of “myths” as the latter implies a judgment or dismissal of concern. Often, concerns from vaccine hesitant groups are not unfounded, for example historical racism in medicine, unknown long-term side effects of vaccination. Similarly, the term “antivaxxer” should not be used for someone who is vaccine hesitant as they lie on different places along the vaccine hesitancy continuum and misrepresenting this is unlikely to be constructive. The feedback stated the concept of “resisting the righting reflex” was crucial. In fact, the feedback requested this concept be introduced earlier in the module. The community group feedback puts words into the mouths of the characters to help bring them to life.

In conclusion, this study suggests that character-driven stories have potential value in online learning about vaccine hesitancy conversations. Further research is needed to establish the nature of their impact on different aspects of learning including patient-related outcomes and the duration of any effect on students’ communication skills.

## Data Availability

QMRO: Using the power of narratives in e-learning for COVID-19 vaccine hesitancy conversations. Self-Confidence scores. https://doi.org/10.17636/10190002 ( Gupta *et al.*, 2023a). The project contains the following underlying data: Self-confidence scores.docx QMRO: Using the power of narratives in e-learning for COVID-19 vaccine hesitancy conversations. Focus Group Transcript anonymous. https://doi.org/10.17636/10190001 ( Gupta *et al.*, 2023b). The project contains the following underlying data: VH transcript.docx QMRO: Pre-course confidence and experience data. https://doi.org/10.17636/10191999 ( Gupta *et al.*, 2023c). The project contains the following underlying data: Easton Pre-course confidence and experience data 2023 Accepted.csv QMRO: Post-course confidence survey. https://doi.org/10.17636/10192000 ( Gupta *et al.*, 2023d) The project contains the following underlying data: Easton Post-course confidence survey 2023 Accepted.csv QMRO: Using the power of narratives in e-learning for COVID-19 vaccine hesitancy conversations. Focus Group Interview Guide. https://doi.org/10.17636/10190985 ( Gupta *et al.*, 2023e). This project contains the following extended data: Easton Using the power of narratives in e-learning for COVID-19 vaccine hesitancy conversations. Focus Group Interview Guide 2023 Accepted.docx Data are available under the terms of the Creative Commons Attribution 4.0 International license (CC-BY 4.0).
