# Supplementary material for: Using the Power of Narratives in E-Learning for COVID-19 Vaccine Hesitancy Conversations: A Mixed Methods Study in Medical Education
Source: MedEdPublish (2016). 2025 May 21;13:310. Originally published 2023 Dec 29. [Version 2] doi: 10.12688/mep.19767.2 (PMC12166355; doi:10.12688/mep.19767.2)
Supplement: Peer review history

# Reviewer report: Reviewer response for version 2

**DOI:** 10.21956/mep.22555.r41879

**Published:** 2025-06-13

**Authors:** Samar Aboulsoud

**Affiliations:** 1 Faculty of Medicine,Internal Medicine Department, Cairo Uiniversity, Cairo, Egypt

I think this version is better than the first one and can be approved.

Have any limitations of the research been acknowledged?

Yes

Is the work clearly and accurately presented and does it cite the current literature?

Partly

If applicable, is the statistical analysis and its interpretation appropriate?

Not applicable

Are all the source data underlying the results available to ensure full reproducibility?

Partly

Is the study design appropriate and is the work technically sound?

Partly

Are the conclusions drawn adequately supported by the results?

Partly

Are sufficient details of methods and analysis provided to allow replication by others?

Partly

Reviewer Expertise:

NA

I confirm that I have read this submission and believe that I have an appropriate level of expertise to confirm that it is of an acceptable scientific standard.

# Reviewer report: Reviewer response for version 1

**DOI:** 10.21956/mep.21181.r40976

**Published:** 2025-01-29

**Authors:** Samar Aboulsoud

**Affiliations:** 1 Faculty of Medicine,Internal Medicine Department, Cairo Uiniversity, Cairo, Egypt

**“Using the power of narratives in e-learning for COVID-19 vaccine hesitancy conversations”**:

 The title of the article is informative but could be improved to better reflect the main message and scope. Including the study design and a hint at the key findings would enhance clarity and attract a broader readership. For instance, explicitly mentioning the target audience or the specific contribution to health professions education (HPE) would help contextualize the work.

 The abstract provides a comprehensive summary of the study, covering the background, methods, results, and conclusions. However, it could benefit from slightly more detail regarding the specific themes identified in the focus group analysis to give readers a clearer sense of the findings.

 The introduction presents a compelling rationale for addressing vaccine hesitancy, especially in the context of the COVID-19 pandemic. The authors appropriately contextualize the need for such an e-learning resource and justify their use of narrative learning theory. However, the section could be strengthened with a more critical analysis of existing literature on narrative learning in healthcare education to firmly establish the knowledge gap this study addresses.

 The methods section provides a solid overview of the resource's development and evaluation process. The integration of narrative learning theory into the design is well-explained, and the involvement of students and community members in co-developing the characters adds credibility and relevance. However, more detail on the survey instrument, particularly its design and validation, would enhance reproducibility. Additionally, a larger and more diverse sample for the focus group might improve the study's generalizability and strengthen the findings.

 The results are clearly presented and indicate an improvement in students’ confidence in having conversations with vaccine-hesitant patients. The thematic analysis of the focus group is well-articulated, identifying key benefits such as memory retention, relatability, and emotional connection. However, the article could further elaborate on how these outcomes align with the theoretical underpinnings of narrative learning to enhance the interpretation of findings.

 The discussion appropriately links the results to existing literature, particularly regarding the benefits of narrative-driven education in healthcare. The authors provide meaningful implications for practice, emphasizing the potential of e-learning to address urgent healthcare challenges. However, the discussion would benefit from a deeper exploration of the limitations, including the small sample size and the lack of patient-related outcome data. Suggestions for future research are mentioned but could be more specific, particularly regarding the kinds of outcomes or metrics that should be prioritized in subsequent studies.

**Typos:**

 There is missing text in the character description in Figure 3 

**Strengths:**

1. **Timely and Relevant Topic:** The focus on COVID-19 vaccine hesitancy addresses an urgent public health challenge.
2. **Innovative Pedagogical Approach:** The use of narrative learning theory and character-driven storytelling is a novel method to enhance engagement and retention.
3. **Rigorous Evaluation:** Combining pre/post confidence assessments and focus group feedback provides a robust mixed-methods evaluation.
4. **Practical Application:** The resource has direct clinical relevance, preparing students for real-world conversations with patients.

**Suggestions for Improvement**

1. **Strengthen Literature Context**: Provide a more robust review of narrative learning in healthcare education to enhance the introduction.
2. **Expand Methodological Details**: Include greater detail on the survey design and focus group participant selection to improve transparency and reproducibility.
3. **Discuss Limitations More Fully**: Acknowledge the small sample size and the absence of patient-centered outcomes in greater depth, with suggestions for addressing these in future work.
4. **Theoretical Integration**: Explicitly link findings (e.g., improved memory, relatability) to narrative learning theory to provide a stronger theoretical foundation.
5. **Future Research**: Offer specific recommendations for further study, such as exploring patient perspectives or evaluating the long-term retention of communication skills.

Overall, the article makes a valuable contribution to health professions education by demonstrating the potential of narrative-based e-learning to address vaccine hesitancy conversations. It effectively bridges theory and practice, offering insights that can inform future educational strategies in similar contexts. With refinements to methodological details and a deeper engagement with theoretical and practical implications, the study could have an even greater impact.

 The references are largely relevant and timely, especially those directly addressing vaccine hesitancy and narrative learning. Some of the older sources, while foundational for narrative learning and focus groups, may require additional context to ensure their applicability to the specific challenges presented by COVID-19 vaccine hesitancy.

Have any limitations of the research been acknowledged?

Yes

Is the work clearly and accurately presented and does it cite the current literature?

Partly

If applicable, is the statistical analysis and its interpretation appropriate?

Not applicable

Are all the source data underlying the results available to ensure full reproducibility?

Partly

Is the study design appropriate and is the work technically sound?

Partly

Are the conclusions drawn adequately supported by the results?

Partly

Are sufficient details of methods and analysis provided to allow replication by others?

Partly

Reviewer Expertise:

CPD, Accreditation, Educational leadership, PGE

I confirm that I have read this submission and believe that I have an appropriate level of expertise to confirm that it is of an acceptable scientific standard, however I have significant reservations, as outlined above.

# Response

**Published:** 2025-04-28

**Authors:** Graham Easton

**Affiliations:** Faculty of Medicine and Dentistry, Queen Mary University of London, London, England, UK

Dear Reviewer, thank you for your comments and suggestions which we feel have improved the paper. Here are our responses below:

**Re title change.** Thank you.

 This is a helpful suggestion. We are not sure if it is possible or advisable to change the title of the paper at this stage, given previous citations etc. If it is possible however, we suggest:  "Using the Power of Narratives in E-Learning for COVID-19 Vaccine Hesitancy Conversations: A Mixed Methods Study in Medical Education" 

**Re results themes in abstract:** Thank you, we agree.We have now provided the full list of themes identified in the focus group analysis:  "The focus group analysis suggests that character-based narratives can promote learning online, particularly through engagement and accessibility, relatability of characters and their stories, improved memory, and emotional connection. "

**Re introduction. **Thank you.  We have added the following text to the introduction, introducing a critical stance and suggesting knowledge gap this study addresses:  “Despite the theoretical promise, there is limited evidence for the benefits of narrative learning approaches on long-term learning outcomes, clinical behaviour or patient outcomes. For example, a systematic review of digital storytelling (which combines traditional storytelling with digital tools) showed minimal impact of patient stories alone on health professionals learning (Moreau, 2018). Developing  models for narrative medicine education and integrating and evaluating diverse storytelling formats, such as the digital storytelling approach we developed in this project, could further enhance student learning experiences”  Additional references needed for this section  Moreau, K.A., Eady, K., Sikora, L. et al. Digital storytelling in health professions education: a systematic review. BMC Med Educ 18, 208 (2018) 

**Regarding the survey instrument**. Thank you.

 This was a simple survey instrument to assess self-confidence. We used accepted best practice in survey item-writing approaches, including use of Likert scales [Artino et al 2014]. However there was no validated survey instrument that we felt entirely appropriate for this specific study.  [ *Artino, A. R., La Rochelle, J. S., Dezee, K. J., & Gehlbach, H. (2014). Developing questionnaires for educational research: AMEE Guide No. 87. Medical Teacher, 36(6), 463–474*.] 

 Regarding the **diversity and small sample size**, we have addressed this in response to Reviewer 1 and have added brief text to limitations section to address this.  Thank you. Regarding the query about the **data reaching saturation and the small sample size:** the novelty and challenges of conducting the study in lockdown during the Covid pandemic [and limited availability of medical students] meant a single focus group was conducted. Pressures on students was an important issue which affected recruitment/sampling – we could only use final year students who had finished their exams. We discuss this elsewhere in paper. Despite this acknowledged limitation, we feel the data was sufficiently rich to share, and that the need for saturation of data became meaningless with only one focus group.  

**Ethnicity/gender **- Thank you. We have added the following text near the start of the results section:   “Whilst we did not formally collect demographic data on ethnicity and gender, overall, we felt the make-up of the focus group broadly reflected the diversity of the medical student body   We have also provided a more robust **review of narrative learning in healthcare in the introduction** **Re results section: **Thank you. In response to reviewer 1, we have added a passage to the discussion section which more explicitly links findings to previous research in narrative learning in medical education. Here is the passage again for clarity:  “Stories are used in various ways in medical education; for example, during ward rounds and clinical case discussions to illustrate diagnostic reasoning and decision-making (Hunter, 1991), or in narrative medicine courses and medical humanities programmes which often use patient stories to foster empathy and reflection (Milota, 2019). Digital storytelling (which combines traditional storytelling with digital tools) is increasingly used to promote patient-centred care patient narratives offering insights into lived experiences (Moreau, 2018) .   There is some limited evidence demonstrating the impact of narrative-based approaches on learning in health professions education . For example, narrative medicine, as explored in a systematic review, has shown positive changes in students’ attitudes, perceptions, attainment of new knowledge and skills, behaviour, and improved awareness of patients’ perspectives (Milota, 2019). Storytelling has been shown to prepare students for uncertainty in clinical practice (Papnagnou, 2021), and can be an effective way for students to learn about medical ethics (Paton, 2021) . In classroom lectures, narratives can provide context, engage learners, and aid memory retention by connecting new knowledge to lived experiences (Easton, 2016).  Digital storytelling has been shown to improve medical students’ critical thinking (Zarei, 2021), although a systematic review of digital storytelling showed minimal impact of patient stories alone on health professionals learning (Moreau, 2018).”     Re **limitations and discussion section**: Thank you. We have now addressed the issue of small sample size in previous responses to Reviewer 1 in limitations section.  he novelty and challenges of conducting the study in lockdown during the Covid pandemic [and limited availability of medical students] meant a single focus group was conducted. Pressures on students was an important issue which affected recruitment/sampling – we could only use final year students who had finished their exams. We discuss this elsewhere in paper.  Despite this acknowledged limitation, we feel the data was sufficiently rich to share, and that the need for saturation of data became meaningless with only one focus group.     We have added some text so relavnt section of  limitations now reads:  “Whilst qualitative studies do not necessarily need large sample sizes to allow collection of in-depth insights, perhaps having two focus groups would have demonstrated a broader range of views. As we used a convenience sample (the challenges of conducting the study in lockdown during the Covid pandemic and limited availability of medical students, meant we only used a single focus group), we cannot be sure how closely our focus group students represent the wider student population, and we should consider volunteer bias ( Heckman, 1990).”  Thank you also for your **suggestion about future studies**. We have added the folllowing passage: “We also think it would be important in future studies to include stronger outcome measures, particularly those relating to the patient perspective and the enduring effect on medical students’ communication skills or empathy, guided outcome measures proposed by Kirkpatrick ( Kirkpatrick et al., 1967) and others”   Thank you again for your comments and suggestions which have been very helpful in developing this second version.

# Reviewer report: Reviewer response for version 1

**DOI:** 10.21956/mep.21181.r40975

**Published:** 2025-01-28

**Authors:** Julie A. Hunt

**Affiliations:** 1 Associate Dean of Clinical Sciences and Professor of Veterinary Medicine, Lincoln Memorial University College of Veterinary Medicine, Harrogate, TN, USA

I am a MEP Advisory Board Member; this review represents my individual viewpoint. While I have performed mixed methods research, I am reviewing this research as a generalist in the topic of narrative learning theory and vaccine hesitancy, not as a specialist.

 The title accurately represents the work.

 The abstract may be improved by identifying the study as mixed methods and by modifying the abstract’s concluding statements to indicate that character-driven stories have potential value in online learning of vaccine hesitancy rather than in the overly broad “online learning environment”.

 The introduction was robustly written.

 The qualitative methods were stronger than the quantitative methods. The focus group and narrative learning theory were well chosen and well justified. Focus group questions should be provided to the reader as an appendix. The pre/post confidence measures are not strong outcome measures. The authors may consider collecting more substantive outcome measures in subsequent studies; they can be guided by stronger outcome measures proposed by Kirkpatrick’s hierarchy (1970), Miller’s pyramid (1990), and Moore et al’s framework (2009).

 In the results section, it would have been helpful to have known the experience levels of the students in terms of COVID vaccines administered, hesitancy encountered, and experience having these conversations prior to the module. These could have been presented in the first paragraph of the results section rather than scattered later in the results section. Some statements could have used additional illustrative quotes.

 In the discussion, the authors state that their themes echo existing literature on narratives in medical education, but they do not discuss where else in medical education these narratives have been used to teach students. Where have these been used, specifically, and what was the outcome? Whose work did the authors’ work build upon? What are the next steps for moving research forward in this line of inquiry?

 In the limitations, the authors discuss qualitative sample size, but they do not state whether their data reached saturation in thematic analysis. This would help the reader to assess the adequacy of the sample size. They also mention the participation of minorities and women. While they reported a gender distribution, they did not report racial or ethnic makeup of their participants. Was this information collected? Could it be reported also?

 Finally, the authors comment that the focus group should not have been facilitated by AG, as AG also created the resource being evaluated. This is a significant conflict of interest, particularly when combined with AG doing the primary qualitative analysis and therefore having the potential to sway the analysis as well. Were the focus group participants aware of this COI? A more thorough discussion of this COI should be added so that the reader can evaluate for themselves the potential for bias.

 This mixed methods study is well grounded in narrative learning theory and evaluates an online, asynchronous learning module on vaccine hesitancy, an important issue for COVID and, increasingly, for other vaccines as well. The authors are commended for using e-learning, asynchronous delivery, motivational interviewing methods, and a thick narrative approach to addressing this important topic.

Have any limitations of the research been acknowledged?

Yes

Is the work clearly and accurately presented and does it cite the current literature?

Yes

If applicable, is the statistical analysis and its interpretation appropriate?

Yes

Are all the source data underlying the results available to ensure full reproducibility?

Partly

Is the study design appropriate and is the work technically sound?

Yes

Are the conclusions drawn adequately supported by the results?

Yes

Are sufficient details of methods and analysis provided to allow replication by others?

Yes

Reviewer Expertise:

Quantitative and mixed methods research in educational outcomes following online learning and simulation-based learning

I confirm that I have read this submission and believe that I have an appropriate level of expertise to confirm that it is of an acceptable scientific standard.

# Response

**Published:** 2025-04-28

**Authors:** Graham Easton

**Affiliations:** Faculty of Medicine and Dentistry, Queen Mary University of London, London, England, UK

Dear Reviewer, thank you for your considered reviewed and your helpful suggestions. We have summaried our responses below: **Re title change**. Thank you. This is a helpful suggestion. We are not sure if it is possible or advisable to change the title of the paper at this stage, given previous citations etc. If it is possible however, we suggest:  "Using the Power of Narratives in E-Learning for COVID-19 Vaccine Hesitancy Conversations: A Mixed Methods Study in Medical Education" **Re results themes in abstract**: Thank you, we agree. We have now provided the full list of themes identified in the focus group analysis:  "The focus group analysis suggests that character-based narratives can promote learning online, particularly through engagement and accessibility, relatability of characters and their stories, improved memory, and emotional connection." Re your suggestion to include link to questionnaire: Thank you. The focus group questions were provided as an appendix at end of paper, under extended data section:  QMRO: Using the power of narratives in e-learning for COVID-19 vaccine hesitancy conversations. Focus Group Interview Guide. https://doi.org/10.17636/10190985 ( Gupta et al., 2023e). **Re introduction.** Thank you.  We have added the following text to the introduction, introducing a critical stance and suggesting knowledge gap this study addresses:  “Despite the theoretical promise, there is limited evidence for the benefits of narrative learning approaches on long-term learning outcomes, clinical behaviour or patient outcomes. For example, a systematic review of digital storytelling (which combines traditional storytelling with digital tools) showed minimal impact of patient stories alone on health professionals learning (Moreau, 2018). Developing  models for narrative medicine education and integrating and evaluating diverse storytelling formats, such as the digital storytelling approach we developed in this project, could further enhance student learning experiences”  Additional references needed for this section  Moreau, K.A., Eady, K., Sikora, L. et al. Digital storytelling in health professions education: a systematic review. BMC Med Educ 18, 208 (2018) **Re: outcome measures**. thank you for the suggestion. We agree that future work could consider additional outcome measures, and we have added this to the discussion section, linked to Reviewer 2 comments along similar lines. We have added the following text to version 2 in the discussion section;  “We also think it would be important in future studies to include stronger outcome measures, particularly those relating to the patient perspective and the enduring effect on medical students’ communication skills or empathy, guided outcome measures proposed by Kirkpatrick ( Kirkpatrick et al., 1967) and others”.  Re providing further detail about students' previous experience.Thank you. We did provide data on student experience and pre-course confidence as an appendix in data section:  QMRO: Pre-course confidence and experience data. https://doi.org/10.17636/10191999 ( Gupta et al., 2023c).  But agree, helpful to provide at start of results section, so we have added a sentence there, summarising what data we have, including the sentence later about some students vaccinated more than 100 patients. Added text:  “In the pre-course survey about students’ confidence and experience of having vaccine hesitancy conversations,  three reported no experience at all, two reported very little experience, two reported some experience, and one reported quite a lot of experience. Some students in the focus group said they had vaccinated more than 100 patients. In terms of self-reported confidence having these conversations, on a scale of 0–10 where 0 is not at all confident, and 10 is totally confident, two students scored 2, three scored 3, one scored 4, one scored 5, one scored 6 and one scored 8.” **Re more detailed review of other narrative uses and evaluations** and how links to our study. Thank you. We have added the following passage to the discussion section:  “Stories are used in various ways in medical education; for example, during ward rounds and clinical case discussions to illustrate diagnostic reasoning and decision-making (Hunter, 1991), or in narrative medicine courses and medical humanities programmes which often use patient stories to foster empathy and reflection (Milota, 2019). Digital storytelling (which combines traditional storytelling with digital tools) is increasingly used to promote patient-centred care patient narratives offering insights into lived experiences (Moreau, 2018) .   There is some limited evidence demonstrating the impact of narrative-based approaches on learning in health professions education . For example, narrative medicine, as explored in a systematic review, has shown positive changes in students’ attitudes, perceptions, attainment of new knowledge and skills, behaviour, and improved awareness of patients’ perspectives (Milota, 2019). Storytelling has been shown to prepare students for uncertainty in clinical practice (Papnagnou, 2021), and can be an effective way for students to learn about medical ethics (Paton, 2021) . In classroom lectures, narratives can provide context, engage learners, and aid memory retention by connecting new knowledge to lived experiences (Easton, 2016).  Digital storytelling has been shown to improve medical students’ critical thinking (Zarei, 2021), although a systematic review of digital storytelling showed minimal impact of patient stories alone on health professionals learning (Moreau, 2018).”  Additional references needed for this section:   Hunter K. Doctors’ Stories - the Narrative Structure of Medical Knowledge. Princeton: Princeton University Press; 1991.  Moreau, K.A., Eady, K., Sikora, L. et al. Digital storytelling in health professions education: a systematic review. BMC Med Educ 18, 208 (2018)  M. M. Milota, G. J. M. W. van Thiel & J. J. M. van Delden (2019) Narrative medicine as a medical education tool: A systematic review, Medical Teacher, 41:7, 802-810  Papanagnou D, Ankam N, Ebbott D, Ziring D. Towards a medical school curriculum for uncertainty in clinical practice. Med Educ Online 2021;26:1972762  Paton A, Kotzee B. The fundamental role of storytelling and practical wisdom in facilitating the ethics education of junior doctors. Health (London) 2021;25:417‑33  Zarei A, Mojtahedzadeh R, Mohammadi A, Sandars J, Hossein Emami SA. Applying digital storytelling in the medical oncology curriculum: Effects on students’ achievement and critical thinking. Ann Med Surg (Lond) 2021;70:102528    Re your comments about **small sample size and saturation of data**.Thank you. Regarding the query about the data reaching saturation and the small sample size: the novelty and challenges of conducting the study in lockdown during the Covid pandemic [and limited availability of medical students] meant a single focus group was conducted. Pressures on students was an important issue which affected recruitment/sampling – we could only use final year students who had finished their exams. We discuss this elsewhere in paper. Despite this acknowledged limitation, we feel the data was sufficiently rich to share, and that the need for saturation of data became meaningless with only one focus group.   We have added some text so relavnt section of  limitations now reads:  “Whilst qualitative studies do not necessarily need large sample sizes to allow collection of in-depth insights, perhaps having two focus groups would have demonstrated a broader range of views. As we used a convenience sample (the challenges of conducting the study in lockdown during the Covid pandemic and limited availability of medical students, meant we only used a single focus group), we cannot be sure how closely our focus group students represent the wider student population, and we should consider volunteer bias ( Heckman, 1990).” **Ethnicity/gender** - Thank you. We have added the following text near the start of the results section:   “Whilst we did not formally collect demographic data on ethnicity and gender, overall, we felt the make-up of the focus group broadly reflected the diversity of the medical student body “ **Re your comments about potential bias of failitator of fous groups**. Thank you. We acknowledge this limitation in Version 1. Students were aware that AG was involved in development of resource. We do agree that theoretically this can introduce bias of course; but recognise that being close to the teaching resource may also bring benefits, and that in our experience it is a common strategy for researchers who developed a teaching session or resource to also run their focus group evaluation.  However, in mitigation, AG was only  minimally involved in developing the actual resource [GE oversaw most of this], we set clear ground rules about encouraging honesty, clarity that AG would not be involved in any of their formal course assessments, and by another author [GE] also independently analysing the data.  We have added text to the limitations section so the relevant passage reads:  “It could be argued that the facilitator for the focus group should have been an impartial individual, as students were aware that AG helped to develop the resource. Guidance recommends that the facilitator should maintain neutrality to avoid bias in participants responses ( ACET Inc, 2011). Although we took care to encourage negative feedback, and students did not seem hesitant to offer these, it is possible students did not contribute all their negative opinions so as not to offend AG. Similarly, there is a risk of moderator bias from accidental leading. However, in mitigation, AG was only  minimally involved in developing the actual resource [GE oversaw most of this], we set clear ground rules about encouraging honesty, clarity that AG would not be involved in any of their formal course assessments, and we ensured another author [GE] also independently analysed the data” Many thanks again for your helpful suggestions and we hope we have addressed your concerns adequately.
